# 

**Published:** 1889-03

**Authors:** 


					﻿Pamphlets, etc., received.
The Sixth Annual Announcement of the Hahneman Hospital College of
San Francisco; session of 1889.
The Report of the Pennsylvania State College for 1887; Agricultural Ex-
perimental Station.
Fourth Annual Report of the Trustees of the Westborough (Mass.) Insane
Hospital.
The Eighteenth Annual Report of the State Homoeopathic Asylum for the
Insane at Middletown, New York.
Is The American Heart Wearing Out ? By J. W. Dowling, M. D. Read
before the N. Y. State Homoeopathic Medical Society, September 11th, 1888.
NOTICE.
All articles for publication, books for review, business co mmunica ileus,ete.,
must be sent to Editors, 1123 Spruce Street, Philadelphia.
Subscribers will please notice that this Journal will be sent until arrears
are paid and it is ordered discontinued.
NOTICE TO SUBSCRIBERS.
The previously announced reduction in the subscription
price of this journal, of fifty cents, to those who remit DI-
R ECTL F to this office, will expire with this month. After
April 1st the charge will be $2.50, cash, and no discount to
any one! A PROMPT remittance will, therefore, save you
FIFTY CENTS and help us.
				

